# Genome-wide cyclin gene evolution in *Arabidopsis* and *Brassica* reveals polyploidization-driven duplication and flowering-time associations

**DOI:** 10.1007/s00438-026-02515-y

**Published:** 2026-09-01

**Authors:** Aldrin Y. Cantila, Sheng Chen, Kadambot H. M. Siddique, Wallace A. Cowling

**Affiliations:** The UWA Institute of Agriculture, The University of Western Australia, Perth, WA 6000 Australia

**Keywords:** Brassicaceae family, Cyclin, Duplication events, Evolutionary mechanism, Pan-genome, Physical clustering

## Abstract

Cyclin genes are plant cell cycle regulators that play essential roles in growth, development, and reproduction. However, the evolutionary dynamics and genomic organization of cyclin genes across the Brassicaceae family remain poorly understood, particularly in the context of allotetraploid genome evolution. Here, we investigated the diversity, expansion mechanisms, and potential functional diversification of cyclin genes across ten Brassicaceae genomes, including four *Arabidopsis* and six *Brassica* species. A total of 1087 cyclin genes representing 23 cyclin types were identified. Comparative genomic analyses revealed that cyclin gene expansion was strongly influenced by polyploidization in *Brassica* species, with 1845 duplication events involving 1063 genes. Whole-genome duplication was the predominant mechanism driving expansion, while both inter- and intra-genomic duplications contributed to gene retention in tetraploid *Brassica* species, with the highest duplication frequency observed in *Brassica juncea*. Across genomes, 120 physical gene clusters were identified, including homogeneous and heterogeneous types. Ortholog analysis between progenitor and allotetraploid species identified 852 orthologous pairs involving 366 genes, indicating extensive conservation following allotetraploid formation. Phylogenetic analysis resolved cyclins into three major clades, while expression-based clustering in *Brassica napus* grouped genes into four major clusters, suggesting functional diversification. Integration of pan-genomic and flowering-time QTL analyses further identified two cyclin genes, *Bna21cycA2* and *Bna113cycD4*, which contain amino acid polymorphisms and represent putative candidate variations potentially associated with flowering-time variation across multiple genomes. These findings provide new insights into the evolutionary expansion, retention, and potential functional divergence of cyclin genes in Brassicaceae and highlight candidate loci for future functional studies and crop improvement.

**Supplementary Information:**

The online version contains supplementary material available at https://doi.org/10.1007/s00438-026-02515-y.

## Introduction

The Brassicaceae family, encompassing over 372 genera and 4060 species, is cultivated as vegetables, edible oil, herbs, spices, condiments and fodder (Warwick 2011; Anjum et al. 2018; Tamokou et al. 2017). Among its member species, the oilseed *Brassica* species (*B. rapa*, *B. napus*, and *B. juncea*) gained global economic importance, ranked as the third-largest source of vegetable oils (Asl and Niazmand 2020). Three tetraploid *Brassica* species and their diploid progenitors form the ‘Triangle of U’ (U 1935), in which polyploidization by interspecific hybridization occurred between the diploid species (*B. rapa*, AA genome with 2n = 20; *B. nigra*, BB genome with 2n = 16; and *B. oleracea*, CC genome with 2n = 18) to form allotetraploid species (*B. juncea*, AABB genome with 2n = 36; *B. carinata*, BBCC genome with 2n = 34; and *B. napus*, AACC genome with 2n = 38). Other species in the Brassicaceae family are significantly used for scientific studies. For instance, *Arabidopsis thaliana* is a model organism in plant genetics (Koornneef and Meinke 2010), *Arabis alpina* in ecological genomics (Wötzel et al. 2022), *Boechera* species in apomixis (Brukhin et al. 2019), *Cardamine hirsuta* in leaf structure and morphology (Gan et al. 2016), and *Lepidium meyenii* in floral structure (Lee et al. 2002).

The rising demand for vegetable proteins, carbohydrates, and oils from Brassicaceae crops highlights the necessity of understanding their reproductive growth and development mechanisms to ensure successful cultivation and to optimize yields in various environments. Reproductive development is a critical stage for the formation of marketable vegetable *Brassica* (for example, in broccoli, cabbage and cauliflower) and for gaining high grain yield in oilseed rape or canola. Knowledge of the genes controlling reproductive traits is crucial for advancing our understanding of these mechanisms. Recent developments in genome sequencing technologies and bioinformatics have greatly expanded the availability of genome sequences in databases (Mohd Saad et al. 2022), which provide a valuable resource for studying reproduction-related genes in crops.

Reproductive development in plants involves a complex transition from vegetative growth to the formation of floral structures. At the onset of floral initiation, several genes function across regulatory pathways and are regarded as “flowering-time regulators” in *A. thaliana* (Bouché et al. 2015; Putterill et al. 2004). These core regulatory genes have been widely used to identify orthologous loci in related species within the Brassicaceae family such as in *B. napus*,* B. rapa*, and *B. oleracea* (Li et al. 2018; Schiessl et al. 2017), *Camelina sativa* (Lily et al. 2021), and *Raphanus sativus* (Wang et al. 2017). Among the orthologs identified in *B. napus*, several cyclin genes were detected (Li et al. 2018). While these genes are primarily known for their role in cell cycle control, their expression in datasets associated with developmental transitions raises their possible involvement in broader reproductive processes. However, their specific contribution to reproductive regulation remains to be clarified.

Numerous studies have highlighted the biological role of cyclin genes, influencing plant developmental processes that occur around the transition to reproductive growth. The loss of function of the cyclin gene *CYCD3* in the *cycd3;1–3* mutant in *A. thaliana*, for instance, resulted in noticeable delay in developmental progression, including flowering under standard growth conditions (Dewitte et al. 2007). In *Antirrhinum majus*, cyclin *D3b* gene expression was modulated during key stages of floral development, including boundary zones between primordia, petal morphogenesis, and in the dorsal anther, influenced by cycloidea activity (Gaudin et al. 2000). In addition, *CYCC1*, a partner of *CDK8* in the Mediator complex, was shown to broadly influence transcription, and its disruption resulted in delayed developmental transitions, phenotypically resembling *cdk8* and *med12* mutants (Hasan 2023). *Arabidopsis MED12* was reported as a modulator of both upstream and downstream components of the key reproductive development genes (Imura et al. 2012), although its role appears multifaceted.

In this study, cyclin genes were identified and their distribution characterized across ten Brassicaceae species. Evolutionary patterns and relationships, including gene duplications types, gene clustering patterns, and phylogenetic groups, were analyzed to gain insights into cyclin gene evolution within the family. Orthologs between *Brassica* progenitors and their polyploid derivatives were also identified. The analysis explored how polyploidization and gene duplication may have shaped cyclin gene expansion and potential functional diversification, and whether specific cyclin variants might be linked to flowering-time variation in *B. napus*. A pan-genomic approach combined with flowering-time-associated quantitative trait loci (QTL) analysis highlighted candidate cyclin genes with potential relevance for crop improvement. In summary, these findings provide a valuable resource for understanding cyclin gene evolution and highlight loci that may contribute to reproductive timing in Brassicaceae species.

## Methods

### Identification of cyclin genes and their gene clusters

The most prominent members of the Brassicaceae family were investigated in this study, including six *Brassica* species in U’s triangle (*B. carinata*, *B. juncea*, *B. napus*, *B. nigra*, *B. oleracea*, and *B. rapa*) (U 1935) and 4 *Arabidopsis* species (*A. arenosa*, *A. lyrata*, *A. suecica*, and *A. thaliana*). Their genomic information, including protein sequences and chromosomal positions for each species, was collated from various publicly available database and publications (Table S1).

To identify cyclin genes, 49 well-characterized *A. thaliana* cyclin genes curated in a previous study (Zhang et al. 2014) were used as reference sequences. These genes have also served as a basis for identifying cyclin genes in *B. rapa* (Zulfiqar et al. 2022) and other plant species (Meng et al. 2020; Zhao et al. 2022a, b). The reference protein sequences were used to identify homologous genes across ten Brassicaceae genomes via BLASTp (Altschul et al. 1997) with compositionally adjusted substitution matrices (Altschul et al. 2005) using the NCBI platform (https://blast.ncbi.nlm.nih.gov/Blast.cgi; accessed 30 May 2026). Candidate homologs were initially retained using an E-value threshold of ≤ 0.001 (Pearson 2013). The resulting putative cyclin proteins were further validated by screening against the Pfam database (Finn et al. 2014) using Hidden Markov Models (HMMs) (Eddy 1998) implemented through the GenomeNet MOTIF platform (https://www.genome.jp/tools/motif/MOTIF.html; accessed 31 May 2026). To further validate homology in *Brassica* genomes, each *Brassica* cyclin gene was subjected to reciprocal BLASTp against the *A. thaliana* proteome, and only those sequences that reciprocally returned the original *Arabidopsis* cyclin genes as the top hit were retained as true homologs. In addition, this study used a standardized custom nomenclature system that assigned the identified cyclin genes based on their closest corresponding homologs among the 49 reference *A. thaliana* cyclin genes.

Gene clusters were defined when two or more cyclin genes were located within a 200 kb region on the same chromosome. Homogeneous clusters consisted of genes belonging to the same gene type, whereas heterogeneous clusters contained genes from different types (Cantila et al. 2022; Jupe et al. 2012).

### Gene duplication and ortholog analyses of cyclin genes

To identify and classify duplication events among cyclin genes, DupGen_finder (Qiao et al. 2019) was used. DupGen_finder categorises gene duplications into five types based on genomic locations and sequence similarity: tandem, proximal, transposed, dispersed, and whole-genome duplication (WGD). All cyclin protein-coding gene models from each species were used as input, with genomic coordinates extracted from the respective genome annotations.

The output for each species included a list of duplication pairs, each with the two gene identifiers, their cyclin subtypes, genomic locations, E-value, and the assigned duplication type. Duplication pairs were further classified as same-type when the two cyclin genes belonged to the same cyclin subtype (e.g., cycA2-cycA2) or cross-type when they belonged to different subtypes (e.g., cycA2-cycB1). The total number of duplication pairs per species, the number of distinct genes involved, and the counts of each duplication type and same-type/cross-type category were calculated. Another type of duplication in the *Brassica* allotetraploid A, B, and C subgenomes (excluding genes located on contigs, scaffolds, or other unplaced sequences) was also recorded. Intra-genomic duplications were defined as duplications occurring within the same subgenome while inter-genomic duplications referred to duplication events occurring between the A, B, and C subgenomes.

Ortholog analysis was performed using OrthoFinder v2.5.5 (Emms and Kelly 2019). Cyclin protein sequences from the diploid progenitor species and their corresponding allotetraploid species were included in the analysis with each pair of species (progenitor vs. allotetraploid), one-to-one orthologous pairs were extracted from the results. In *Brassica* allotetraploids, orthologs were assigned to their respective A, B, or C subgenomes based on genome annotations, enabling assessment of progenitor genome contributions and patterns of gene retention following polyploidization. Orthologs originating from a diploid progenitor were considered retained if they were located within the expected corresponding subgenomes (e.g., *B. rapa* genes in the A subgenome, *B. nigra* genes in the B subgenome, and *B. oleracea* genes in the C subgenome). Conversely, orthologs located in a non-corresponding subgenome were classified as exchanged. The numbers and percentages of retained and exchanged orthologs were subsequently calculated for each progenitor-allotetraploid combination.

### Phylogenetic and transcriptomics-based analyses of cyclin genes

To examine the evolutionary relationships and expression divergence of cyclin genes, both sequence-based phylogenetic analysis and transcriptomics-based clustering were performed. For the sequence-based phylogenetic analysis, cyclin protein sequences from *Arabidopsis* and six *Brassica* species were retrieved. After removing identical sequences, the remaining sequences were retained for subsequent analyses. These sequences were aligned using Multiple Alignment using Fast Fourier Transform (Katoh and Standley 2013), and poorly aligned regions were trimmed prior to phylogenetic reconstruction. Phylogenetic analysis was conducted using IQ-TREE v3.1.2 (Minh et al. 2020), with the best-fit substitution model selected by ModelFinder (Kalyaanamoorthy et al. 2017) under the Bayesian Information Criterion. The Q.PFAM+R3 model was identified as the optimal substitution model. A maximum likelihood (ML) phylogenetic tree was reconstructed using nearest-neighbour interchange branch swapping with 1,000 ultrafast bootstrap replicates (Hoang et al. 2018) to assess branch support. The resulting phylogenetic tree was visualized and annotated using the Interactive Tree of Life (iTOL) platform (Letunic and Bork 2024).

For the transcriptomics-based analysis, previously published RNA-seq datasets (in transcript per million, TPM) from reproductive development stages were utilized and were retrieved from the Sequence Read Archive (SRA) (Table S2) via BrassicaEBD (Chao et al. 2020) https://brassica.biodb.org/downloads; accessed 30 May 2026. As the Darmor-bzh v5 genome is the most widely used reference for *B. napus* transcriptomic studies, gene identifiers were harmonised to the corresponding models in the updated Darmor-bzh v10 assembly based on the mapping framework established by Rousseau-Gueutin et al. (2020). This framework integrated reciprocal best-hit protein alignments using DIAMOND and BLASTp, together with synteny refinement and stringent sequence similarity criteria.

Unsupervised hierarchical clustering was performed to investigate expression-based relationships among cyclin genes using a TPM expression matrix. Genes with zero variance across samples were first removed, as correlation-based distance measures cannot be computed for constant expression profiles. The remaining expression values were transformed using log₂ (TPM + 1) (Harris et al. 2020) to stabilise variance and appropriately handle zero expression values, following standard RNA-seq normalisation practices. Pairwise gene distances were calculated using correlation distance (1-Pearson correlation coefficient), enabling clustering based on similarity in expression patterns rather than absolute expression levels. Hierarchical clustering was then conducted using Ward’s minimum variance linkage method to minimise within-cluster variance during cluster formation. All analyses were implemented in Python version 3.13 (Python Software Foundation, 2025) using the SciPy library (Virtanen et al. 2020). The tree was exported in Newick format using the scipy.cluster.hierarchy.to_tree function with a custom recursive traversal and was subsequently visualized using iTOL (Letunic and Bork 2024). This approach allowed direct comparison between expression-based clustering and sequence-based phylogenetic relationships of the cyclin types.

### Investigating cyclin genes associated with flowering time in *Brassica napus*

Flowering time, a key determinant of environmental adaptation and yield stability, was selected as the target trait. SNPs from the *Brassica* 60 K Illumina Infinium array, previously reported to be significantly associated with flowering time (Raman et al. 2019; Xu et al. 2015; Helal et al. 2021) with their positions in genome *B. napus* Darmor-bzh v10 (Vollrath et al. 2021). A sensitivity analysis was performed using sliding window sizes of 50, 100, 200, and 500 kb, together with gene positional information in *B. napus*, to support subsequent QTL delineation. Cyclin genes colocalizing with these QTL were subjected to pan-genomic analysis using eight *B. napus* genomes with contrasting flowering phenotypes, obtained from a previous study (Song et al. 2020). These genotypes included two early-flowering (Westar and No2127), two late-flowering (Quinta and Tapidor), and four intermediate-flowering (Gangan, Shengli, ZS11, and Zheyou7).

Protein sequences of QTL-colocalizing cyclins were queried against the above eight genomes using BLASTp via the BnaOmics (Cui et al. 2023). Homologous proteins were aligned using Multiple Sequence Comparison by Log-Expectation (MUSCLE) in Geneious prime (Kearse et al. 2012), and phylogenetic relationships were inferred using the Neighbor-Joining method with 10,000 bootstrap replicates. Structural variation, including presence/absence and sequence deletions, was assessed across genomes to identify polymorphisms potentially associated with flowering time.

## Results

### Identification and distribution of cyclin genes

**Fig. 1:**
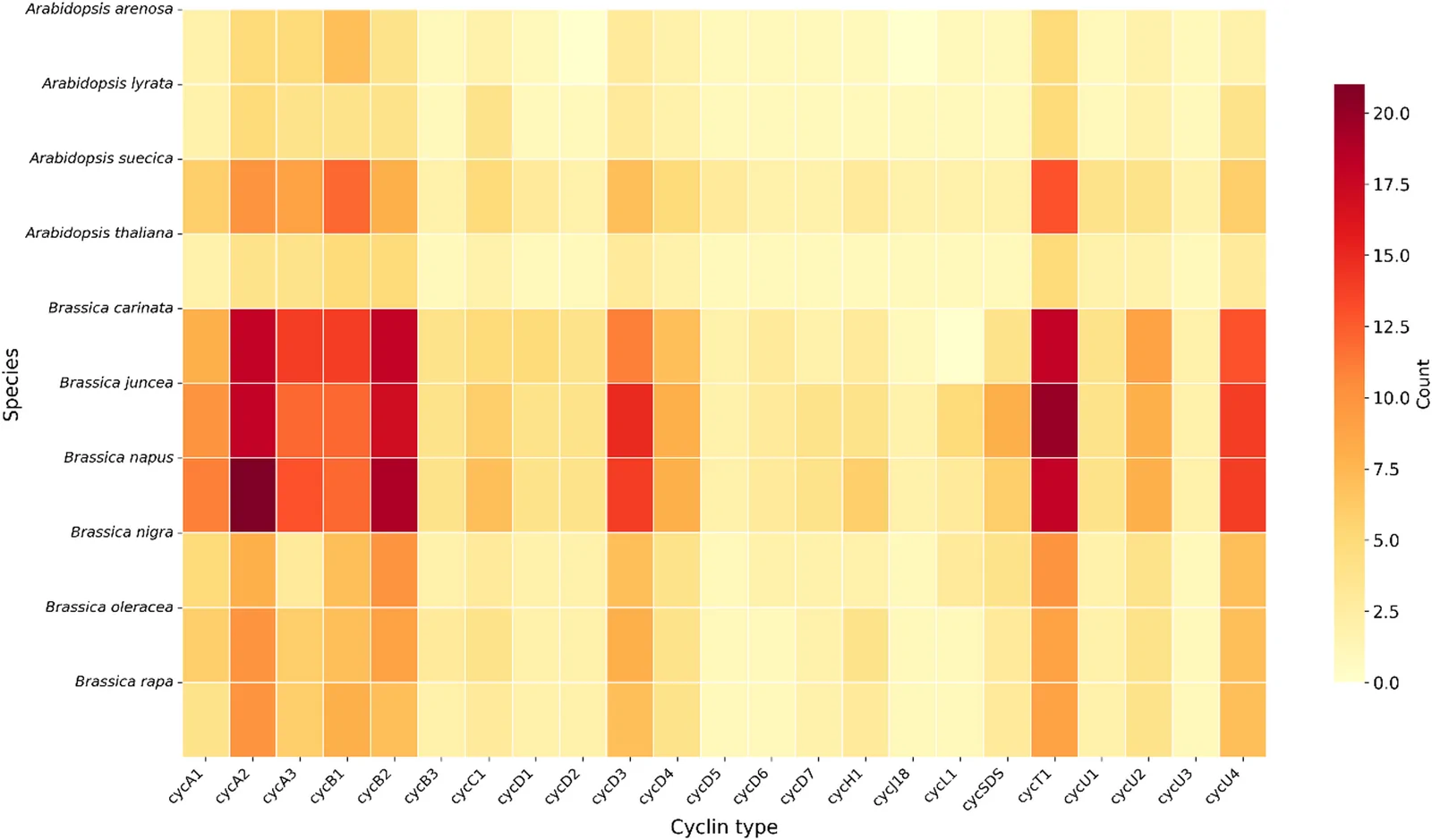
Heat map of cyclin gene counts per type in ten Brassicaceae species

A total of 1123 putative cyclin homologs were initially identified using 49 *A. thaliana* cyclins as queries (Table S3). These candidate cyclin homologs were further evaluated for the presence of conserved cyclin domains using Pfam analysis, which confirmed 1087 proteins containing cyclin domains across four *Arabidopsis* species and six *Brassica* crop species (Fig. 1, Tables S3-S4). For the *Brassica* species, 823 cyclin proteins were further validated by reciprocal BLASTp against the *A. thaliana* proteome and classified as reciprocally supported homologs (Tables S3, S5).

Among the 23 cyclin types detected, cycA (241 genes) and cycD (225 genes) were the most numerous, followed by cycB (213 genes), cycU (164 genes), and cycT (112 genes). The cycD gene type exhibited the highest subtype diversity, consisting of cycD1 (25 genes), cycD2 (22), cycD3 (78), cycD4 (46), cycD5 (15), cycD6 (18), and cycD7 (21) (Fig. 1, Table S3).

Across the four *Arabidopsis* species, cyclin counts ranged from 49 to 114, with an average of 66 genes per species (Fig. 1). The six *Brassica* species showed broader variation, ranging from 90 to 189, with an overall average of 137.2 cyclins per species (Fig. 1, Table S3). The six diploid species (*A. arenosa*, *A. lyrata*, *A. thaliana*, *B. nigra*, *B. oleracea*, *B. rapa*) displayed similar totals, averaging 71.5 cyclin genes per species. In contrast, the four allotetraploid species (*A. suecica*, *B. carinata*, *B. juncea*, *B. napus*) contained substantially higher counts, ranging from 114 to 189, with an average of 164.5 cyclin genes, clearly exceeding the diploid average (Fig. 1, Table S3).

### Gene duplication in cyclin genes

**Fig. 2:**
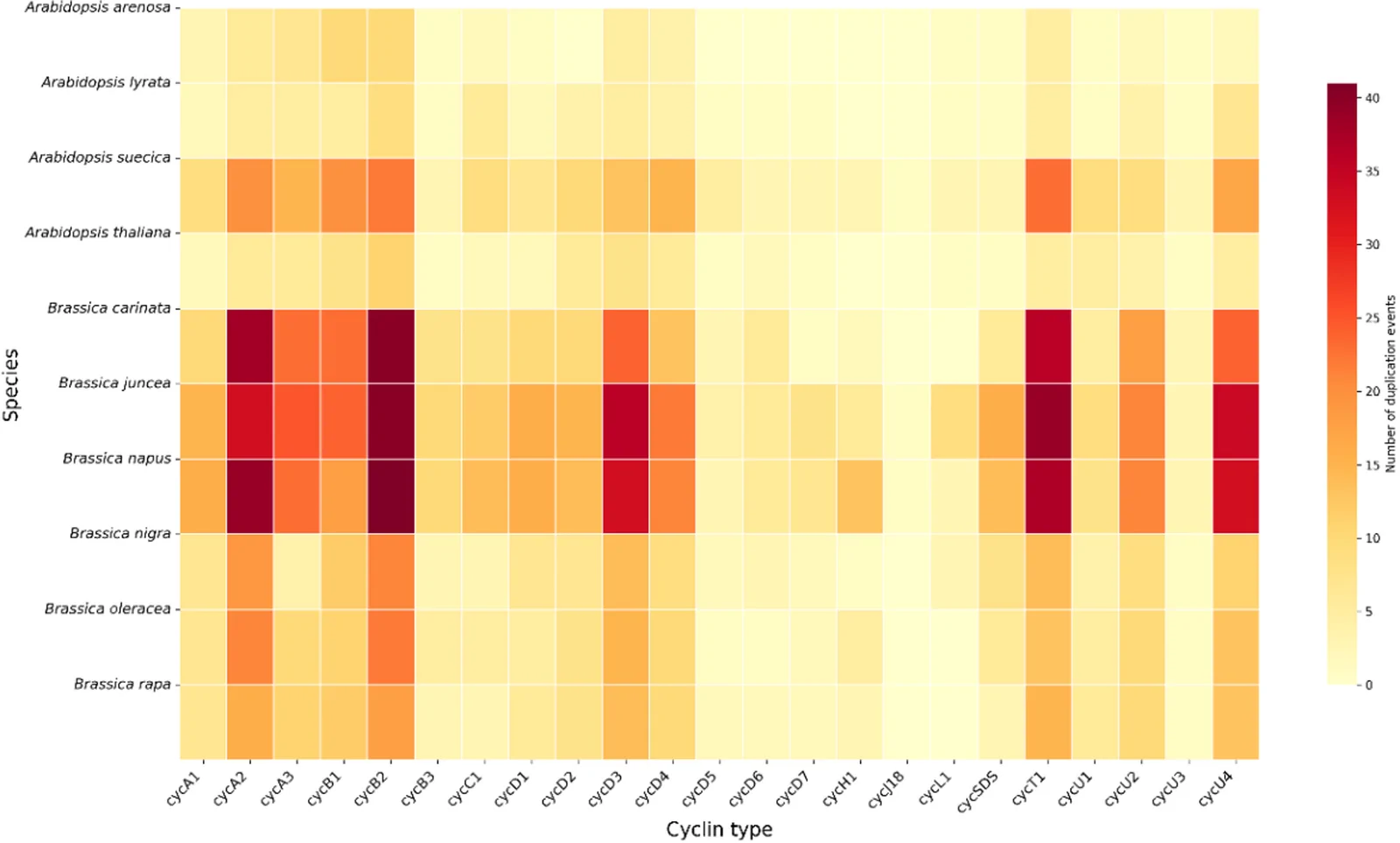
Heat map of duplication event counts among cyclin types across ten Brassicaceae species

**Fig. 3:**
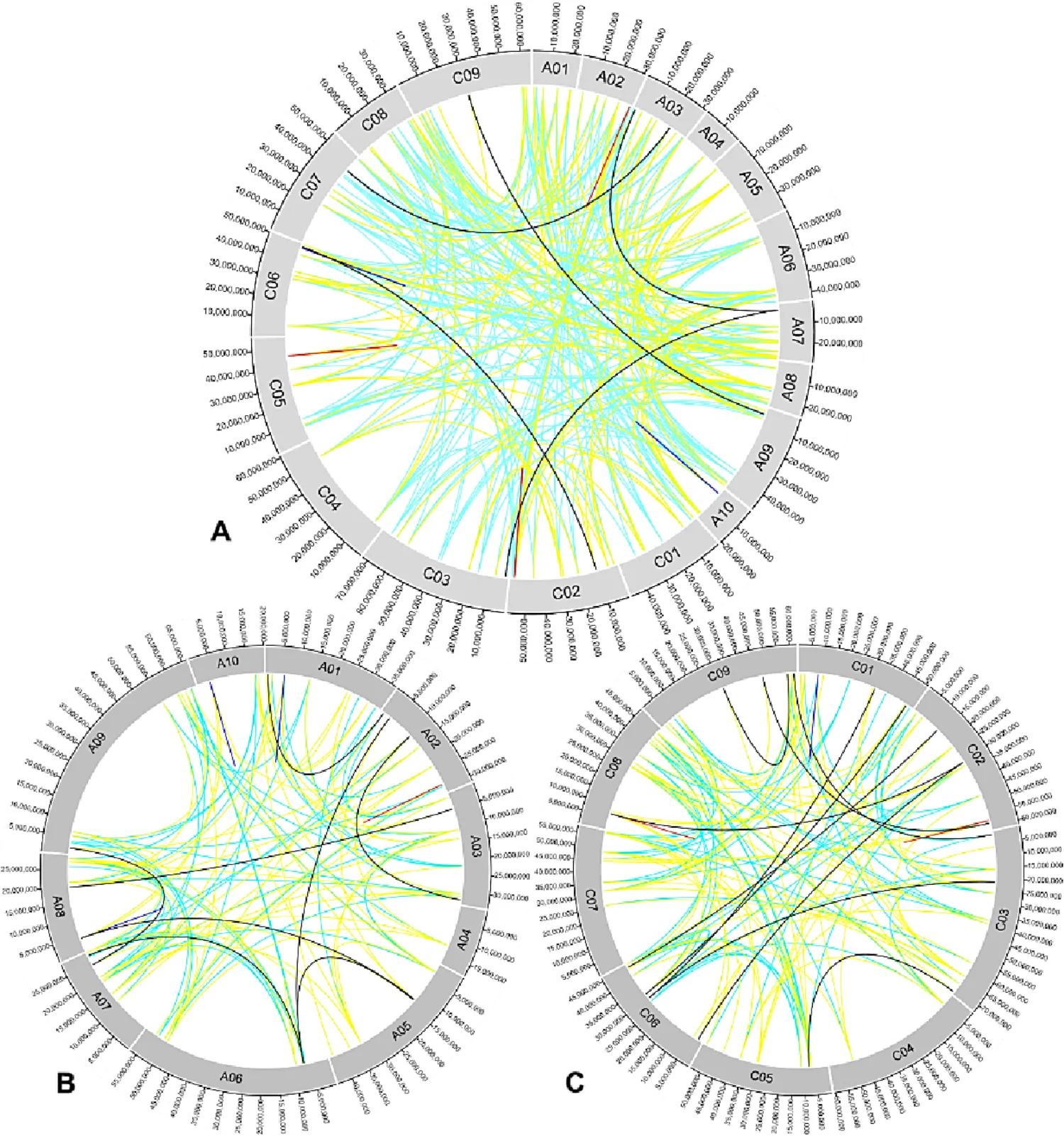
Duplication of cyclin genes in the tetraploid *Brassica napus* (**A**) and its two ancestral species, *B. rapa* (**B**) and *B. oleracea* (**C**), visualized using a Circos plot. The outer track displays chromosomal positions of cyclin genes for each chromosome (middle green track). The inner track shows connecting lines between duplicated gene pairs, with each connection representing a single duplication event. Connecting line colors indicate different duplication types: cyan = whole-genome duplication, yellow = dispersed, blue = proximal, red = tandem, and black = transposed. Numbers shown on the outer track indicate base pair positions along the chromosomes

Across 10 genomes, a total of 1,845 duplication events were identified involving 1063 cyclin genes (Fig. 2, Table S6). The highest number of duplication events was observed in *B. juncea*, with 377 events involving 185 genes, followed by *B. napus* (372 events involving 186 genes), *B. carinata* (299 events involving 167 genes), and *A. suecica* (197 events involving 114 genes ). The remaining species, all of which are diploids, contained fewer than 160 duplication events and fewer than 100 cyclin genes involved in duplication events each. For example, the diploid progenitors of *B. napus*, *B. rapa* and *B. oleracea*, contained fewer duplication events, with 154 events involving 87 genes and 140 events involving 94 genes, respectively (Fig. 3). On average, tetraploids showed 311.3 duplication events involving 163 genes, whereas diploids showed an average of 100.0 duplication events involving 68.5 genes (Fig. 2, Table S6). When comparing genera, *Brassica* species displayed a higher frequency of gene duplication than *Arabidopsis*. On average, *Brassica* species had 247.3 duplication events involving 134.8 cyclin genes, while *Arabidopsis* species showed only 90.3 duplication events involving 63.5 genes (Table S6).

In terms of cyclin gene types, cycB2 showed the highest level of duplication, with 234 duplication events, followed by cycA2 and cycT1 which exhibited 203 and 192 duplication events, respectively (Fig. 2, Table S6). Intermediate levels of duplication were observed for cycT1, cycU4, cycA3, cycD4, and cycU2, each showing at least 100 duplication (Fig. 2, Table S6). In contrast, the remaining cyclin types displayed relatively limited duplication, with fewer than 100 duplication events. Cross-type cyclin duplications were also identified (Table S6). Among these, the cycD2-cycD4 pairing exhibited the highest number of duplication events (31), followed by cycB2-cycB3 and cycU1-cycU2, each with 20 duplication events, and cycD1-cycD2 with 18 duplication events. All remaining cross-type pairings exhibited fewer than 15 duplication events each.

In the duplication result, 969 events were classified as WGD, whereas 730 events were identified as dispersed, 20 as proximal, 51 as tandem, and 75 as transposed duplications (Table S6). In *Brassica* species, WGD duplications accounted for at least 45% of all duplication events (e.g., 68% in *B. juncea*), while in *Arabidopsis* species this proportion varied, with *A. suecica* showing 42% WGD and *A. thaliana* showing 22% WGD. The highest numbers of WGD duplication events were observed in *B. juncea* (254 events) and *B. napus* (254 events), followed by *B. carinata* (151 events) and *A. suecica* (83 events). The remaining species each exhibited fewer than 70 WGD duplication events. Tandem duplication events were relatively rare. *A. suecica* showed the highest number, with 19 tandem duplications, followed by *B. carinata* with 11 events, while all other species exhibited fewer than seven tandem duplication events.

Additional duplication categories, namely intra-genomic and inter-genomic duplications as presented in Table S6, were examined exclusively in allotetraploid *Brassica* species due to their well-defined A, B, and C subgenomes. In *B. carinata*, a total of 274 duplication events were identified after excluding pairs involving unplaced scaffolds or contigs. Among these, 44 intra-genomic pairs occurred within the B subgenome and 76 intra-genomic pairs within the C subgenome, accounting for 120 intra-genomic events, while the remaining 154 pairs represented inter-genomic duplications. In *B. juncea*, 359 duplicated gene pairs were identified, including 66 intra-genomic pairs within the A subgenome and 81 intra-genomic pairs within the B subgenome, totalling 147 intra-genomic events and 212 inter-genomic events. Similarly, in *B. napus*, 363 duplicated gene pairs were detected after excluding unplaced sequences, with 83 intra-genomic pairs within the A subgenome and 66 intra-genomic pairs within the C subgenome, giving 149 intra-genomic and 214 inter-genomic duplications.

### Gene clustering of cyclin genes in genomes

A total of 120 cyclin gene clusters were identified across the genomes, comprising 263 genes (Table S7). *A. suecica* exhibited the highest number of clusters (22 clusters involving 51 genes), followed by *B. juncea* (20 clusters involving 41 genes), *B. carinata* (19 clusters involving 39 genes), and *B. napus* (19 clusters involving 45 genes). The remaining species each contained fewer than 10 clusters, with fewer than 20 genes involved per species. Of these 120 clusters, 88 were homogeneous cyclin clusters, while the remaining 32 clusters were heterogeneous. CycA formed the largest number of homogeneous clusters (24 clusters involving 55 genes), followed by cycU (19 clusters involving 38 genes), cycC (14 clusters involving 32 genes), and cycT (13 clusters involving 28 genes). The other cyclin types formed fewer than 10 clusters each, with fewer than 25 genes involved per type.

### Orthologous pairs of cyclin genes across diploid progenitors and allotetraploids

**Fig. 4:**
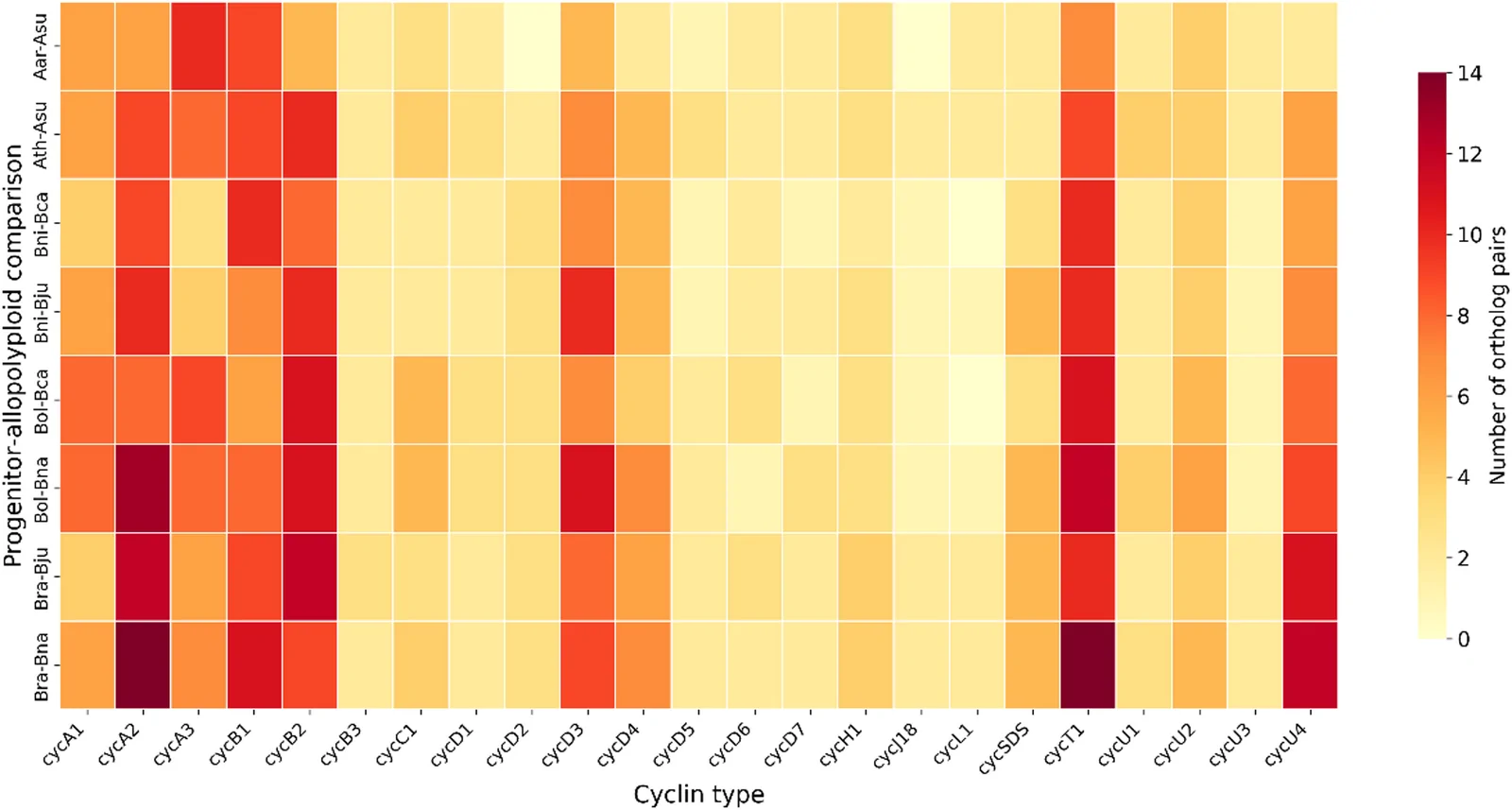
Heat map of ortholog pair counts between diploid progenitors and allotetraploids across cyclin types in Brassicaceae

**Fig. 5:**
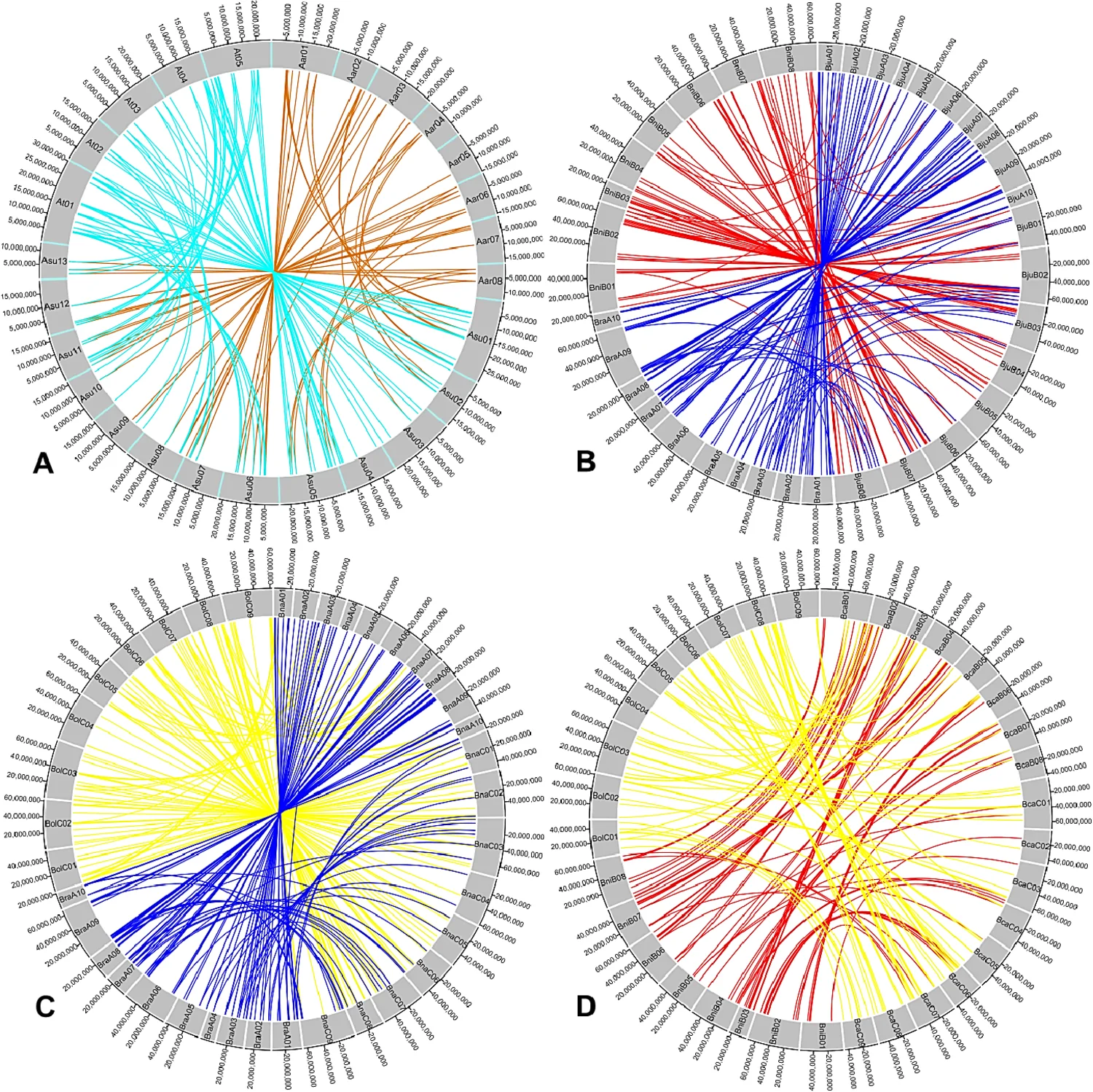
Orthologous relationships of cyclin genes between progenitor and allotetraploid species are visualized using Circos plots: *Arabidopsis arenosa* and *A. thaliana* to *A. suecica* (**A**); *Brassica rapa* and *B. nigra* to *B. juncea* (**B**); *B. rapa* and *B. oleracea* to *B. napus* (**C**); and *B. nigra* and *B. oleracea* to *B. carinata* (**D**). The outer track displays chromosomal positions of cyclin genes for each chromosome (middle green track). The inner track shows colored connecting lines between orthologous gene pairs. Connecting line colors indicate progenitor origin: cyan = *A. thaliana*, orange = *A. arenosa*, blue = *B. rapa*, red = *B. nigra*, and yellow = *B. oleracea*. Numbers shown on the outer track indicate base pair positions along the chromosomes

A total of 852 ortholog pairs were identified across all progenitor-allotetraploid comparisons, involving 366 genes (Figs. 4 and 5, Table S8). Among *Brassica* comparisons, the highest number of ortholog pairs from diploid progenitors to allotetraploids was observed for *B. rapa* to *B. napus* (129 pairs, 90 genes), followed by *B. oleracea* to *B. napus* (127 pairs, 94 genes), *B. rapa* to *B. juncea* (117 pairs, 88 genes), *B. oleracea* to *B. carinata* (106 pairs, 81 genes), *B. nigra* to *B. juncea* (100 pairs, 83 genes), and *B. nigra* to *B. carinata* (88 pairs, 77 genes). In the *Arabidopsis* lineage, 106 ortholog pairs were identified from *A. thaliana* to *A. suecica* (50 genes), followed by 79 ortholog pairs from *A. arenosa* to *A. suecica* (45 genes).

The subgenomic retention and exchange of cyclin orthologs from diploid progenitors to *Brassica* allotetraploids were presented in Table S9. In *B. napus*, orthologs from the A-genome progenitor *B. rapa* showed 67.7% retention on the expected A subgenome (86 out of 127 pairs) and 32.2% exchange to the C subgenome, while those from the C-genome progenitor *B. oleracea* exhibited 68.8% retention on the C subgenome (86 out of 125 pairs) and 31.2% exchange to the A subgenome. In *B. juncea*, *B. rapa* orthologs were retained on the A subgenome in 73.9% of cases (85 out of 115 pairs), with 26.0% exchanged to the B subgenome; *B. nigra* orthologs showed higher retention on the B subgenome (87.5%; 84 out of 96 pairs) and only 12.5% exchange to the A subgenome. In *B. carinata*, *B. nigra* orthologs were retained on the B subgenome in 66.6% of cases (54 out of 81 pairs) and exchanged to the C subgenome in 33.3% of cases, whereas *B. oleracea* orthologs showed 70.4% retention on the C subgenome (69 out of 98 pairs) and 29.5% exchange to the B subgenome. Overall, retention rates varied from 66.6% to 87.5% depending on the progenitor-allopolyploid combination, with the lowest retention observed for *B. nigra* in *B. carinata* (66.6%) and the highest for *B. nigra* in *B. juncea* (87.5%). Exchange events (orthologs found on a non-expected subgenome) occurred in all comparisons, with frequencies between 12.5% and 33.3%.

In terms of cyclin types, cycT1 (83 pairs, 38 genes) and cycA2 (81 pairs, 36 progenitor genes) were the most abundant, followed by cycB2 (76 pairs, 34 genes), cycB1 (69 pairs, 32 genes), cycD3 (64 pairs, 28 genes), cycU4 (61 pairs, 24 genes), cycA3 (55 pairs, 24 genes), and cycA1 (48 pairs, 19 genes) (Fig. 4, Table S8). Among D-type cyclins, cycD3 showed the highest retention (64 pairs), while cycD4 (41 pairs), cycD2 (20 pairs), cycD1 (19 pairs), cycD6 (17 pairs), cycD7 (15 pairs) and cycD5 (14 pairs) were less frequent (Fig. 4, Table S8). The remaining types (cycC1, cycH1, cycU2, cycU1, cycSDS, cycB3, cycU3, cycJ18, cycL1) each accounted for fewer than 40 ortholog pairs and involved fewer than 20 progenitor genes (Fig. 4, Table S8). These results, obtained with a robust phylogeny-based orthology inference, confirm that most cyclin genes are retained in *Brassica* allotetraploids and that retention patterns differ substantially among species pairs and subgenomes.

### Phylogenetic analysis of the cyclin genes in *Arabidopsis* and *Brassica* species

**Fig. 6:**
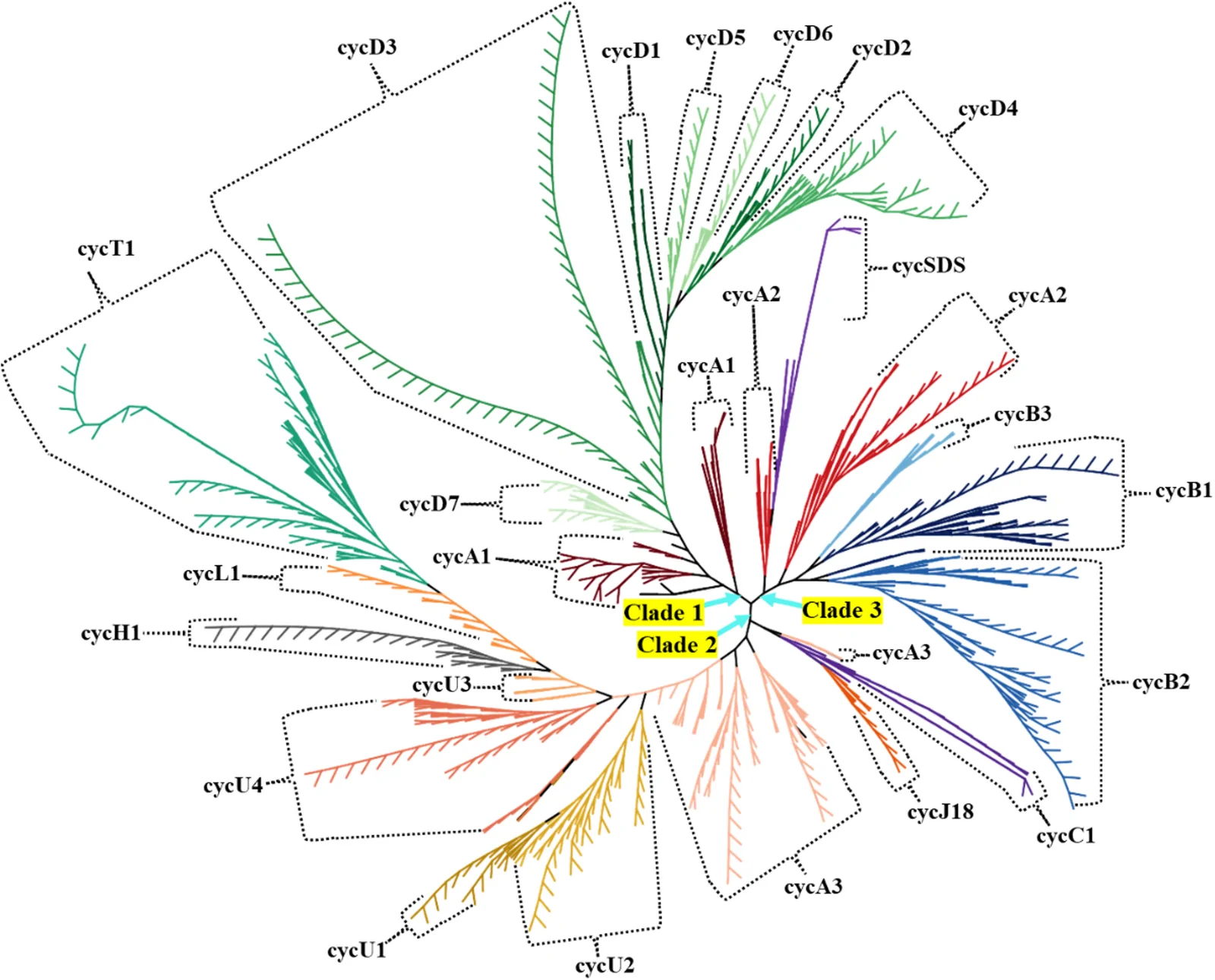
Sequence-based phylogeny of 1079 cyclin genes from ten Brassicaceae genomes, illustrating evolutionary relationships among cyclin types

After quality filtering, 1079 out of 1087 cyclin protein sequences were used for the ML phylogenetic tree construction. The phylogenetic tree resolved three major clades with distinct cyclin-type compositions (Fig. 6). Clade 1 contained 281 members and was predominantly composed of cycD types (225 members), together with a smaller number of cycA1 (56 members). The distribution of cycD types within this clade included cycD3 (78), cycD4 (46), cycD1 (25), cycD2 (22), cycD7 (21), cycD6 (18), and cycD5 (15). Clade 2 was the largest group, comprising 401 members. It contained a mixture of cycA2 and multiple cycB types, including cycA2 (103), cycB2 (101), cycB1 (87), and cycB3 (24), as well as cycC1 (41), cycSDS (33), and cycJ18 (12). Clade 3 comprised 397 members and represented the most heterogeneous group. It included cycT1 (112), cycU4 (77), cycA3 (75), cycU2 (47), cycH1 (28), cycU1 (26), cycL1 (18), and cycU3 (14).

### Transcriptome-expression abundance dendrogram analysis of the cyclin genes in *Brassica napus*

Of the 189 identified *B. napus* cyclin genes, 15 genes annotated in the Darmor v5 genome assembly had no corresponding genes in Darmor v10 and were excluded from subsequent analysis (Table S10). The remaining 174 genes were evaluated for expression across 22 RNA-seq TPM datasets, of which seven genes showed no detectable expression across all datasets and were excluded from expression pattern analyses (Table S10). Therefore, 167 *B. napus* cyclin genes were retained for clustering analysis.

**Fig. 7:**
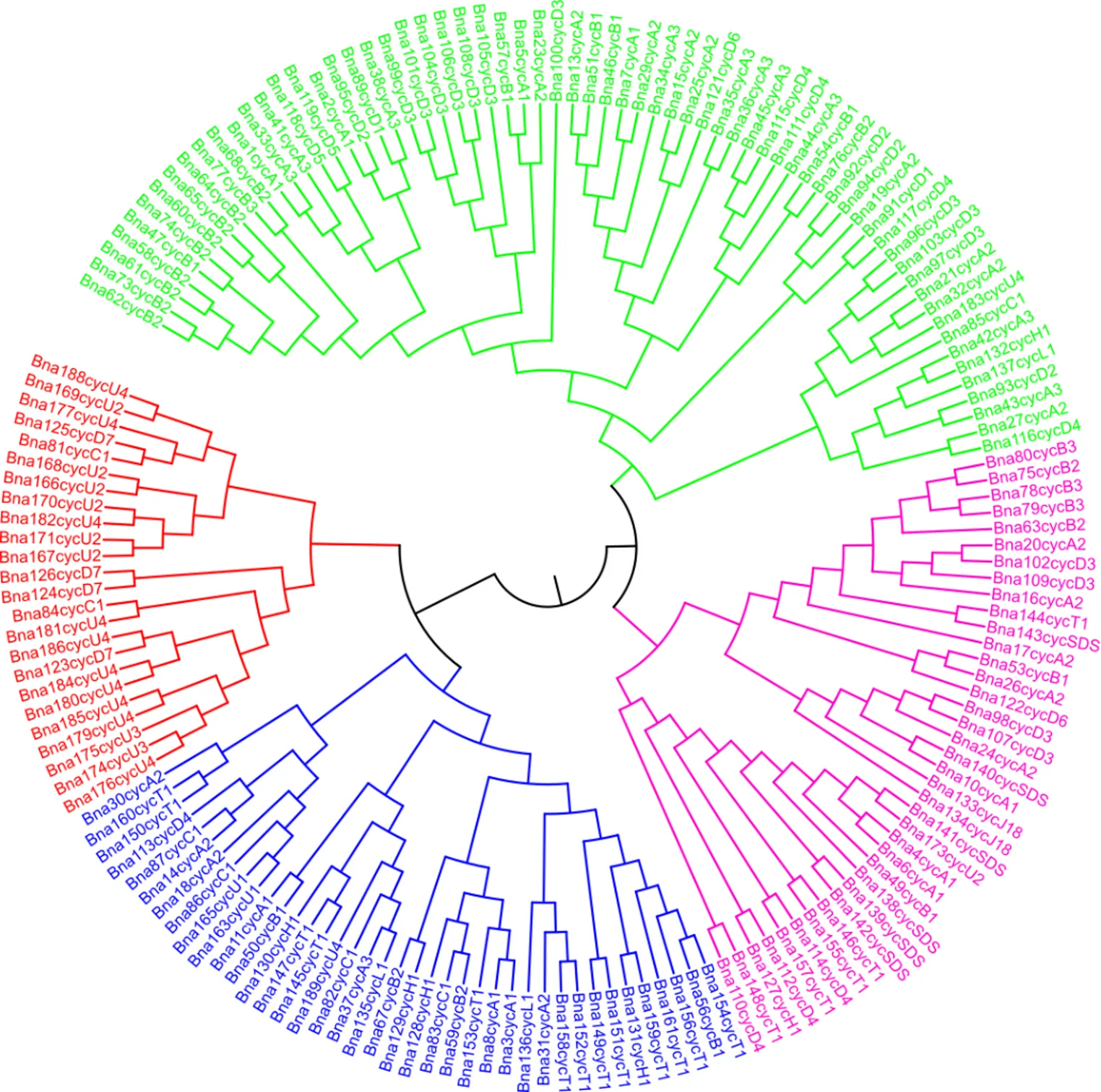
Hierarchical clustering of 167 cyclin genes based on log₂-transformed (TPM + 1) expression values across 22 RNA-seq datasets representing reproductive developmental stages in *Brassica napus*. Genes were grouped into four clusters (I-IV) according to similarities in their expression profiles. Clusters I, II, III, and IV are indicated in green, purple, blue, and red, respectively

Hierarchical clustering of their expression profiles identified four distinct expression clusters (Fig. 7), with uneven cluster sizes: Cluster 1 (66 genes), Cluster 2 (38 genes), Cluster 3 (39 genes), and Cluster 4 (24 genes). Cluster 1 was the largest and most diverse, comprising a broad representation of cycA-, B-, and D-types, including 23 cycA, 16 cycB, and 22 cycD genes, along with a small number of other cyclins (one each of cycC1, cycH1, cycL1, and cycU4). This broad pattern suggests that multiple cyclin families may share coordinated transcriptional regulation during reproductive development. Cluster 2 was characterized by the presence of six cycSDS and five cycT1 genes, together with smaller numbers of cycA, cycB, and cycD3 genes. Cluster 3 was dominated by cycT1 genes (13 members), along with several cycC1, cycH1, cycL1, and cycU1 genes. In contrast, Cluster 4 was highly distinct and consisted predominantly of U-type cyclins (six cycU2, two cycU3, and ten cycU4), with only a few cycC1 and cycD7 genes.

### Linking cyclin genes to flowering time phenotypes with pan-genomic insights

A total of 174 SNPs previously reported to be significantly associated with flowering time in *B. napus* were compiled and used to define QTL regions. These QTL intervals were delineated using a 50 kb window based on sensitivity analysis and subsequently used to identify colocalising cyclin genes (Table S12). Of the 189 cyclin genes in *B. napus* here, five of them, Bna21cycA2, Bna84cycC1, Bna85cycC1, Bna99cycD3 and Bna113cycD4, fell within the QTL intervals and were therefore selected for pan-genomic comparison across eight *B. napus* cultivars representing early, intermediate, and late flowering types (Table S12-13).

**Fig. 8:**
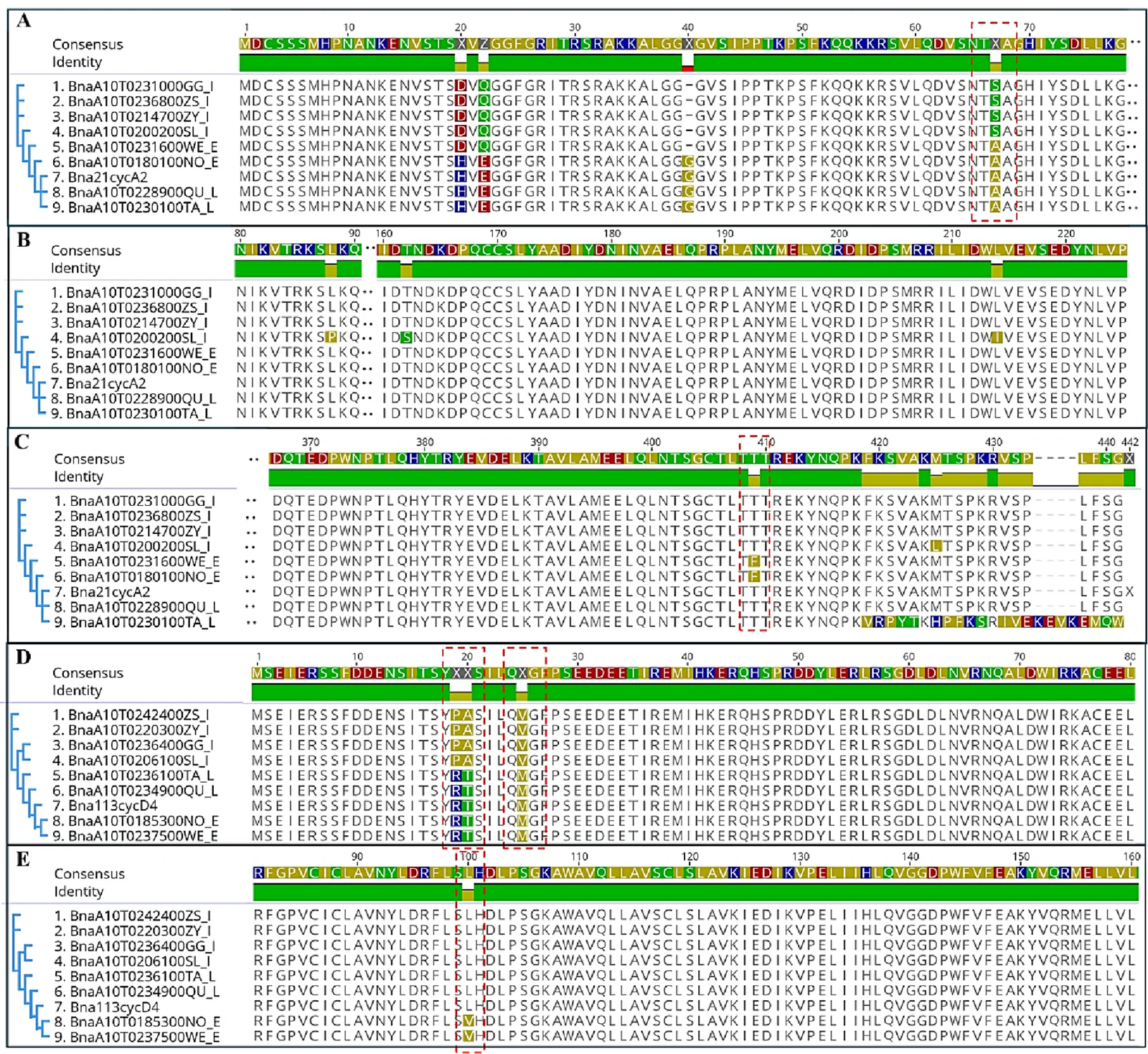
Amino acid polymorphisms distinguishing flowering-time groups in *Brassica napus*. Gene name suffixes indicate cultivar identity (ZS = ZS11, ZY = Zheyou7, GG = Gangan, TA = Tapidor, SL = Shengli, QU = Quinta, NO = No2127, WE = Westar) and flowering phenotype (E = early, I = intermediate, L = late) for Bna21cycA2 and Bna113cycD4. Numbers above the alignment denote amino acid positions based on the multiple sequence alignment. Panels A-C correspond to Bna21cycA2, and panels D-E correspond to Bna113cycD4. Red dashed boxes highlight diagnostic amino acid polymorphisms that differentiate flowering-time phenotypes

Analysis of the five cyclin genes and their homologs was shown in Table S13 and Fig. 8. Some genes showed complete sequence conservation (100% query coverage and 100% identity) in specific cultivars, including Bna99cycD3 in ZS11, Westar, Tapidor, Shengli, Quinta, Gangan, and Zheyou7, Bna113cycD4 in Tapidor and Quinta, Bna21cycA2 in Quinta, Bna85cycC1 in Zheyou7, Westar, Shengli, and Gangan, highlighting strong conservation of gene structure across these genomes. On the other hand, several cyclin genes also showed high similarity across several *B. napus* genomes, including Bna21cycA2 in No2127, Zheyou7, ZS11, Gangan, Westar, Shengli, and Tapidor, Bna113cycD4 in Westar, No2127, Zheyou7, ZS11, Shengli, and Gangan, and Bna99cycD3 in Zheyou7. Other cyclin genes here were less similar across genomes with lower query coverage (45–100%) and identity values ranging from 70.8 to 100% like Bna84cycC1 in Gangan, Zheyou7, Shengli, Westar, and Tapidor, and Bna85cycC1 in Tapidor, Quinta, No2127, and ZS11. Also, no BLASTp hits were found in Bna84cycC1 in Quinta, ZS11, No2127. These results suggest structural differences such as truncations, copy number changes, and gene absence. These patterns provide useful candidates for exploring variation associated with flowering behaviour.

Among the five genes, Bna21cycA2 and Bna113cycD4 and their homologs showed consistent phylogenetic patterns across the eight *B. napus* genomes. Notably, the early-flowering genotypes Westar and No2127 were always grouped within the same clades for both genes, a pattern that was not observed from the other genomes. This suggests a shared and distinct sequence variation associated with early flowering. In Bna21cycA2, both Westar and No2127 carry a phenylalanine (F) at amino acid position 411, whereas all other genomes possess a threonine (T) at this site (Fig. 8). Similarly, in Bna113cycD4, Westar and No2127 share a valine (V) at position 100, while the remaining genomes contain a leucine (L). These consistent amino acid substitutions provide strong candidates for developing molecular markers specific to early-flowering genotypes.

For the intermediate-flowering group (Zheyou7, ZS11, Shengli, and Gangan), distinct polymorphisms were also observed. In Bna21cycA2, these genomes show unique amino acid variant at position 67. In Bna113cycD4, intermediate genotypes differ at positions 19, 20, and 25 relative to both early and late groups. For the late-flowering genotypes, particularly Quinta and Tapidor, gene-specific molecular markers could also be designed. For Bna21cycA2, a forward primer may be positioned around the 20th amino acid region, with a reverse primer designed near the 400th amino acid region. Similarly, for Bna113cycD4, a marker could be developed using a forward primer around the 19th or 20th amino acid position and a reverse primer near the 100th amino acid region.

Together, these patterns suggest that these genes may contain sequence variants that distinguish early, mid, and late flowering types; however, their potential role in flowering time requires further verification through genetic mapping or functional studies.

## Discussion

This study investigated the physical occurrence and evolutionary mechanism of cell cycle protein cyclin genes in ten published genomes within the Brassicaceae family. Significant variation was observed in both the number and composition of cyclin genes, including differences in dispersed, proximal, tandem, transposed, WGD duplication types, inter- and intra-genomic duplication among *Brassica* allotetraploids, and the organization of cyclin genes into distinct clusters across species, genera, and ploidy levels.

Cyclin genes are highly abundant due to their diverse and essential roles in plant development (Chaubet-Gigot 2000; Inzé and De Veylder 2006; Patterson et al. 2021; Kumar and Larkin 2017). The prevalence of cyclin-A, B, and D in the Brassicaceae family likely reflects both their critical roles in regulating the plant cell cycle and their preferential retention during evolution. A-type cyclins are tightly regulated during the cell cycle, with sequential expression patterns observed during cell-cycle progression, and specific members such as *Nicta; CYCA3;2* playing key roles at the interface of cell division and differentiation (Reichheld et al. 1996; Yu et al. 2003). Cyclin-B is pivotal in controlling the G2-to-M phase transition during key developmental stages (Weingartner et al. 2003; Fobert et al. 1994; Giménez-Abián et al. 2002). Numerous studies have underscored the importance of A-, B-, and D-type cyclins in both mitotic cell cycle and mitotic growth (Chaubet-Gigot 2000; Ito 2000; Meijer and Murray 2000). Beyond their cell cycle roles, cycA is implicated in auxin signaling and meristem formation (Roudier et al. 2003), and these cyclins interact with CDKs to regulate additional plant processes (Roudier et al. 2000). CycB, for instance, governs embryonic development, seed size (Zhao et al. 2022a, b), and promotes seed formation (Niu et al. 2020), as well as playing a role in microtubule organization (Romeiro Motta et al. 2022). CycD is specifically linked to cell division in the stomatal lineage of the hypocotyl epidermis (Kono et al. 2007).

Among the two genera studied, *Brassica* exhibited the highest number of cyclin genes, largely driven by the presence of three allotetraploid species, which increased overall gene counts. This expansion is primarily attributed to a more recent and extensive whole-genome triplication event in the *Brassica* lineage, which was not experienced by the *Arabidopsis* lineage (Chalhoub et al. 2014). Across species, allotetraploids consistently contained more cyclin genes than diploids, with elevated numbers of duplicated and clustered genes that likely contribute to the retention of essential cell cycle regulators and enhanced regulatory flexibility, underscoring their importance in plant growth, development, and reproduction (Cheng et al. 2018; Almeida-Silva and Van de Peer 2025).

The expansion of cyclin genes in Brassicaceae has been largely driven by multiple rounds of WGD, followed by diversification through additional duplication modes. Consistent with previous studies, the majority of duplicated cyclin genes originated from WGD events, reflecting the widespread occurrence of paleopolyploidization across land plant evolution (Panchy et al. 2016; Sankoff and Zheng 2018; Shi et al. 2026). Subsequent tandem, proximal, and dispersed duplications likely contributed to the further expansion, functional diversification, and retention of cyclin family members.

The observed expansion patterns of cyclin genes in the six cultivated *Brassica* species are intrinsically linked to the lineage-specific whole-genome triplication (WGT) event, which provided the initial genomic material for a three- to six-fold increase in gene copies in diploid and allotetraploid species, respectively (Chalhoub et al. 2014; Cheng et al. 2014; Song et al. 2021). However, gene copy numbers alone do not fully explain the current distribution of cyclin genes. Following WGT in *Brassica*, extensive gene fractionation and differential gene loss have played a major role in shaping gene family evolution (Cheng et al. 2018). In particular, selection acting on gene dosage is expected to influence the retention or loss of duplicated genes, especially those involved in dosage-sensitive networks and protein complexes. The gene balance hypothesis suggests that selection maintains dosage-sensitive genes within balanced interaction networks to preserve protein stoichiometry, as disruptions in copy number or expression, such as those caused by homoeologous exchanges in allopolyploids, can create genomic imbalance and constrain gene retention and expression patterns (Bird et al. 2023). Such processes are consistent with observations that certain genes are preferentially retained as single copies due to strong functional constraints, while others are more frequently lost following duplication events (De Smet et al. 2013). In these allotetraploid species *B. napus*, *B. juncea*, and *B. carinata*, extensive genome reshuffling and homoeologous exchange have further shaped cyclin gene distribution, leading to gene relocation and copy number variation has produced the complex and non-uniform cyclin gene distribution observed in this study. These processes can generate gene copies that are physically distant within chromosomes yet remain highly similar in sequence, thereby resembling dispersed duplication patterns (Hurgobin et al. 2018). Furthermore, inter- and intra-genomic duplications in *Brassica* allotetraploids reflect their evolutionary origin following hybridization between distinct diploid progenitors, forming the A and C subgenomes under the 'Triangle of U' model. Subsequent polyploidization was followed by extensive chromosome rearrangements and homoeologous exchanges, further contributing to subgenome restructuring and gene retention dynamics and changes (Bird et al. 2023; Yang et al. 2016; Yim et al. 2022).

Polyploidization in Brassicaceae is well established to increase gene copy numbers compared to diploid relatives, through chromosome doubling or tripling (Chalhoub et al. 2014; Song et al. 2021; Kagale et al. 2014). Interspecific hybridization and subsequent genome duplication have contributed to fixed heterozygosity in allopolyploid species (Takahagi et al. 2018; Chen 2010, 2013). This is supported by comparative studies in other plant families, such as *Arachis*, where polyploid species were found to possess a greater abundance of cyclin genes than their diploid counterparts (Gokul et al. 2023).

Homogeneous clusters likely arise from tandem duplication events and may provide functional redundancy or facilitate coordinated regulation of gene expression (Razin et al. 2021). Heterogeneous clusters, in contrast, arise through segmental duplication, ectopic recombination, translocations, and gene transpositions (Meyers et al. 2003; Richly et al. 2002; Perazzolli et al. 2014; Marone et al. 2013). Functional roles of clustered cyclin genes with diverse domains remain to be fully elucidated.

Phylogenetic analysis revealed that cyclin genes cluster into distinct clades corresponding to functional classes, indicating early diversification and strong sequence conservation across Brassicaceae (Jia et al. 2014; Menges et al. 2007; Bulankova et al. 2013; Meskiene et al. 1995). The grouping of cycD types and cycA1 members within Clade I, cycA2 and cycB types within Clade II, and cycC, cycH, cycP (including cycU), cycSDS, cycL, cycT, and cycJ18 within Clade III has been consistently reported in phylogenetic studies of cyclin genes across plant species (Wang et al. 2017; Jia et al. 2014), suggesting that the major cyclin lineages originated early in plant evolution and have retained their functional identities despite subsequent gene duplication and diversification events. On the other hand, transcriptomics-based phylogen showed substantial divergence in expression patterns among duplicates, which indicates that regulatory changes, rather than coding sequence evolution, drive functional diversification (Casneuf et al. 2006). The phylogenetic relationships are based on multi-species sequence and structural features, whereas the expression clusters are species-specific and functionally defined; thus, a one-to-one correspondence between the two is not expected. Larger expression-based clusters likely reflect coordinated regulation during reproductive development, while smaller clusters may support specialized or condition-specific roles. These complementary analyses collectively highlight how cyclin genes are both evolutionarily conserved and functionally diversified within Brassicaceae.

Linking cyclin genes to an economically important trait such as flowering time in canola, our results show that Bna21cycA2 and Bna113cycD4, which co-localize with previously reported flowering-time QTLs, were identified using a 50 kb window for QTL delineation. This threshold is supported by linkage disequilibrium decay estimates in *B. napus*, including a genome-wide half-decay of less than 45 kb (Rahman et al. 2022) and chromosome-specific decay ranging from 7 to 68 kb (Horvath et al. 2020). Both genes harbor sequence variations that correlate with flowering phenotypes in *B. napus*. Early-flowering cultivars, Westar and No2127, cluster together and share distinctive amino acid substitutions absent in other cultivars, whereas intermediate-flowering cultivars display unique variants. These observations suggest that these genes are putative candidates potentially associated with flowering-time variation in *B. napus*. However, their roles in flowering-time regulation require further verification through genetic mapping and functional validation.

These results highlight the multifunctional roles of conserved cyclin domains. Beyond their classical involvement in cell cycle regulation, cyclins appear to participate in broader developmental and environmental networks, particularly during reproductive development in *B. napus*. This study lays the groundwork for functional validation and suggests that manipulating cyclin genes could offer a strategy to control key reproductive traits, such as flowering, in Brassicaceae crops. It also underscores the value of pan-genomic investigations to uncover novel regulatory elements for breeding climate-resilient and high-yielding varieties.

## Supplementary Information

Below is the link to the electronic supplementary material.


Supplementary Material 1


## Data Availability

All data generated or analysed during this study are included in this published article and its supplementary information files.

## References

[CR1] Almeida-Silva F, Van de Peer Y (2025) Gene expression divergence following gene and genome duplications in spatially resolved plant transcriptomes. Plant Cell 37(10). 10.1093/plcell/koaf243 41092097

[CR2] Altschul SF, Madden TL, Schäffer AA, Zhang J, Zhang Z, Miller W, Lipman DJ (1997) Gapped BLAST and PSI-BLAST: a new generation of protein database search programs. Nucleic Acids Res 25(17):3389–3402. 10.1093/nar/25.17.3389 9254694 PMC146917

[CR3] Altschul SF, Wootton JC, Gertz EM, Agarwala R, Morgulis A, Schäffer AA, Yu YK (2005) Protein database searches using compositionally adjusted substitution matrices. FEBS J 272(20):5101–5109. 10.1111/j.1742-4658.2005.04945.x 16218944 PMC1343503

[CR4] Anjum NA, Gill SS, Dhankher OP, Jimenez JF, Tuteja N (2018) Editorial: the brassicaceae-agri-horticultural and environmental perspectives. Front Plant Sci 9:1141. 10.3389/fpls.2018.01141 30210507 PMC6120433

[CR5] Asl JP, Niazmand R (2020) Bioactive phytochemicals from rapeseed (*Brassica napus*) oil processing by-products. In: Ramadan Hassanien MF (ed) Bioactive phytochemicals from vegetable oil and oilseed processing by-products. Springer International Publishing, Cham, pp 1–22. 10.1007/978-3-030-63961-7_3-1

[CR6] Bird KA, Pires JC, VanBuren R, Xiong Z, Edger PP (2023) Dosage-sensitivity shapes how genes transcriptionally respond to allopolyploidy and homoeologous exchange in resynthesized *Brassica napus*. Genetics 225(1):iyad114. 10.1093/genetics/iyad114 37338008 PMC10471226

[CR7] Bouché F, Lobet G, Tocquin P, Périlleux C (2015) FLOR-ID: an interactive database of flowering-time gene networks in *Arabidopsis thaliana*. Nucleic Acids Res 44(D1):D1167–D1171. 10.1093/nar/gkv1054 26476447 PMC4702789

[CR8] Brukhin V, Osadtchiy JV, Florez-Rueda AM, Smetanin D, Bakin E, Nobre MS, Grossniklaus U (2019) The boechera genus as a resource for apomixis research. Front Plant Sci. 10.3389/fpls.2019.00392 PMC6454215 31001306

[CR9] Bulankova P, Akimcheva S, Fellner N, Riha K (2013) Identification of Arabidopsis meiotic cyclins reveals functional diversification among plant cyclin genes. PLoS Genet 9(5):e1003508. 10.1371/journal.pgen.1003508 23671425 PMC3649987

[CR10] Cantila AY, Neik TX, Tirnaz S, Thomas WJW, Bayer PE, Edwards D, Batley J (2022) Mining of cloned disease resistance gene homologs (CDRHs) in *Brassica* species and *Arabidopsis thaliana*. Biology (Basel). 10.3390/biology11060821 PMC9220128 35741342

[CR98] Casneuf T, De Bodt S, Raes J, Maere S, Van de Peer Y (2006) Nonrandom divergence of gene expression following gene and genome duplications in the flowering plant Arabidopsis thaliana. Genome Biol 7(2):R13. 10.1186/gb-2006-7-2-r13 PMC1431724 16507168

[CR11] Chalhoub B, Denoeud F, Liu S, Parkin IA, Tang H, Wang X, Chiquet J, Belcram H, Tong C, Samans B, Correa M, Da Silva C, Just J, Falentin C, Koh CS, Le Clainche I, Bernard M, Bento P, Noel B, Labadie K, Alberti A, Charles M, Arnaud D, Guo H, Daviaud C, Alamery S, Jabbari K, Zhao M, Edger PP, Chelaifa H, Tack D, Lassalle G, Mestiri I, Schnel N, Le Paslier MC, Fan G, Renault V, Bayer PE, Golicz AA, Manoli S, Lee TH, Thi VH, Chalabi S, Hu Q, Fan C, Tollenaere R, Lu Y, Battail C, Shen J, Sidebottom CH, Wang X, Canaguier A, Chauveau A, Berard A, Deniot G, Guan M, Liu Z, Sun F, Lim YP, Lyons E, Town CD, Bancroft I, Wang X, Meng J, Ma J, Pires JC, King GJ, Brunel D, Delourme R, Renard M, Aury JM, Adams KL, Batley J, Snowdon RJ, Tost J, Edwards D, Zhou Y, Hua W, Sharpe AG, Paterson AH, Guan C, Wincker P (2014) Plant genetics. Early allopolyploid evolution in the post-Neolithic Brassica napus oilseed genome. Science 345(6199):950–953. 10.1126/science.1253435 25146293

[CR12] Chao H, Li T, Luo C, Huang H, Ruan Y, Li X, Niu Y, Fan Y, Sun W, Zhang K, Li J, Qu C, Lu K (2020) BrassicaEDB: a gene expression database for brassica crops. Int J Mol Sci 21(16):5831. 10.3390/ijms21165831 32823802 PMC7461608

[CR13] Chaubet-Gigot N (2000) Plant A-type cyclins. Plant Mol Biol 43(5–6):659–675. 10.1023/a:1006303100592 11089868

[CR14] Chen ZJ (2010) Molecular mechanisms of polyploidy and hybrid vigor. Trends Plant Sci 15(2):57–71. 10.1016/j.tplants.2009.12.003 20080432 PMC2821985

[CR15] Chen ZJ (2013) Genomic and epigenetic insights into the molecular bases of heterosis. Nat Rev Genet 14(7):471–482. 10.1038/nrg3503 23752794

[CR16] Cheng F, Wu J, Wang X (2014) Genome triplication drove the diversification of *Brassica* plants. Hortic Res 1:14024. 10.1038/hortres.2014.24 26504539 PMC4596316

[CR17] Cheng F, Wu J, Cai X, Liang J, Freeling M, Wang X (2018) Gene retention, fractionation and subgenome differences in polyploid plants. Nat Plants 4(5):258–268. 10.1038/s41477-018-0136-7 29725103

[CR18] Cui X, Hu M, Yao S, Zhang Y, Tang M, Liu L, Cheng X, Tong C, Liu S (2023) BnaOmics: a comprehensive platform combining pan-genome and multi-omics data from (Brassica napus). Plant Commun. 10.1016/j.xplc.2023.100609 PMC10504585 37098652

[CR21] De Smet R, Adams KL, Vandepoele K, Van Montagu MC, Maere S, Van de Peer Y (2013) Convergent gene loss following gene and genome duplications creates single-copy families in flowering plants. Proc Natl Acad Sci U S A 110(8):2898–2903. 10.1073/pnas.1300127110 23382190 PMC3581894

[CR22] Dewitte W, Scofield S, Alcasabas AA, Maughan SC, Menges M, Braun N, Collins C, Nieuwland J, Prinsen E, Sundaresan V, Murray JAH (2007) *Arabidopsis* CYCD3 D-type cyclins link cell proliferation and endocycles and are rate-limiting for cytokinin responses. Proc Natl Acad Sci U S A 104(36):14537–14542. 10.1073/pnas.0704166104 17726100 PMC1964848

[CR19] Eddy SR (1998) Profile hidden Markov models. Bioinformatics 14(9):755–763. 10.1093/bioinformatics/14.9.755 9918945

[CR20] Emms DM, Kelly S (2019) OrthoFinder: phylogenetic orthology inference for comparative genomics. Genome Biol 20(1):238. 10.1186/s13059-019-1832-y 31727128 PMC6857279

[CR23] Finn RD, Bateman A, Clements J, Coggill P, Eberhardt RY, Eddy SR, Heger A, Hetherington K, Holm L, Mistry J, Sonnhammer EL, Tate J, Punta M (2014) Pfam: the protein families database. Nucleic Acids Res 42:222–230. 10.1093/nar/gkt1223 PMC3965110 24288371

[CR24] Fobert PR, Coen ES, Murphy GJ, Doonan JH (1994) Patterns of cell division revealed by transcriptional regulation of genes during the cell cycle in plants. EMBO J 13(3):616–624. 10.1002/j.1460-2075.1994.tb06299.x 8313906 PMC394851

[CR25] Gan X, Hay A, Kwantes M, Haberer G, Hallab A, Ioio RD, Hofhuis H, Pieper B, Cartolano M, Neumann U (2016) The Cardamine hirsuta genome offers insight into the evolution of morphological diversity. Nat Plants 2:16167. 10.1038/nplants.2016.167 27797353 PMC8826541

[CR26] Gaudin V, Lunness PA, Fobert PR, Towers M, Riou-Khamlichi C, Murray JA, Coen E, Doonan JH (2000) The expression of D-cyclin genes defines distinct developmental zones in snapdragon apical meristems and is locally regulated by the Cycloidea gene. Plant Physiol 122(4):1137–1148. 10.1104/pp.122.4.1137 10759509 PMC58948

[CR27] Giménez-Abián JF, Weingartner M, Binarova P, Clarke DJ, Anthony RG, Calderini O, Heberle-Bors E, Moreno Díaz, de la Espina S, Bögre L, De la Torre C (2002) A topoisomerase II-dependent checkpoint in G2-phase plant cells can be bypassed by ectopic expression of mitotic cyclin B2. Cell Cycle 1(3):187–192. 10.4161/cc.1.3.124 12429932

[CR74] Gokul Babu S, Gohil DS, Roy Choudhury DS S (2023) Genome-wide identification, evolutionary and expression analysis of the cyclin-dependent kinase gene family in peanut. BMC Plant Biol 23(1):43. 10.1186/s12870-023-04045-w 36658501 PMC9850575

[CR28] Harris CR, Millman KJ, van der Walt SJ, Gommers R, Virtanen P, Cournapeau D, Wieser E, Taylor J, Berg S, Smith NJ, Kern R, Picus M, Hoyer S, van Kerkwijk MH, Brett M, Haldane A, Del Río JF, Wiebe M, Peterson P, Gérard-Marchant P, Sheppard K, Reddy T, Weckesser W, Abbasi H, Gohlke C, Oliphant TE (2020) Array programming with NumPy. Nature 585(7825):357–362. 10.1038/s41586-020-2649-2 32939066 PMC7759461

[CR29] Hasan A (2023) Genetic and molecular analysis of two new loci controlling flowering in garden pea. Univ Tasmania (thesis). 10.25959/100.00031728

[CR30] Helal MMU, Gill RA, Tang M, Yang L, Hu M, Yang L, Xie M, Zhao C, Cheng X, Zhang Y, Zhang X, Liu S (2021) SNP- and haplotype-based GWAS of flowering-related traits in Brassica napus. Plants 10(11):2475. 10.3390/plants10112475 34834840 PMC8619824

[CR31] Hoang DT, Chernomor O, von Haeseler A, Minh BQ, Vinh LS (2018) UFBoot2: improving the ultrafast bootstrap approximation. Mol Biol Evol 35(2):518–522. 10.1093/molbev/msx281 29077904 PMC5850222

[CR32] Horvath D, Stamm M, Talukder Z, Fiedler J, Horvath A, Horvath G, Chao W, Anderson J (2020) A new diversity panel for winter rapeseed (*Brassica napus* L.) genome-wide association studies. Agronomy 10:2006. 10.3390/agronomy10122006

[CR97] Hurgobin B, Golicz AA, Bayer PE, Chan CK, Tirnaz S, Dolatabadian A, Schiessl SV, Samans B, Montenegro JD, Parkin IAP, Pires JC, Chalhoub B, King GJ, Snowdon R, Batley J, Edwards D (2018) Homoeologous exchange is a major cause of gene presence/absence variation in the amphidiploid Brassica napus. Plant Biotechnol J 16(7):1265-1274. 10.1111/pbi.12867 PMC5999312 29205771

[CR33] Imura Y, Kobayashi Y, Yamamoto S, Furutani M, Tasaka M, Abe M, Araki T (2012) CRYPTIC PRECOCIOUS/MED12 is a novel flowering regulator with multiple target steps in Arabidopsis. Plant Cell Physiol 53(2):287–303. 10.1093/pcp/pcs002 22247249 PMC3278046

[CR34] Inzé D, De Veylder L (2006) Cell cycle regulation in plant development. Annu Rev Genet 40:77–105. 10.1146/annurev.genet.40.110405.090431 17094738

[CR35] Ito M (2000) Factors controlling cyclin B expression. Plant Mol Biol 43(5–6):677–690. 10.1023/a:1006336005587 11089869

[CR36] Jia R-D, Guo C-C, Xu G-X, Shan H-Y, Kong H-Z (2014) Evolution of the cyclin gene family in plants. J Syst Evol 52(5):651–659. 10.1111/jse.12112

[CR37] Jupe F, Pritchard L, Etherington GJ, Mackenzie K, Cock PJ, Wright F, Sharma SK, Bolser D, Bryan GJ, Jones JD, Hein I (2012) Identification and localisation of the NB-LRR gene family within the potato genome. BMC Genomics 13:75. 10.1186/1471-2164-13-75 22336098 PMC3297505

[CR38] Kagale S, Robinson SJ, Nixon J, Xiao R, Huebert T, Condie J, Kessler D, Clarke WE, Edger PP, Links MG, Sharpe AG, Parkin IA (2014) Polyploid evolution of the Brassicaceae during the Cenozoic era. Plant Cell 26(7):2777–2791. 10.1105/tpc.114.126391 25035408 PMC4145113

[CR39] Kalyaanamoorthy S, Minh BQ, Wong TKF, von Haeseler A, Jermiin LS (2017) ModelFinder: fast model selection for accurate phylogenetic estimates. Nat Methods 14(6):587–589. 10.1038/nmeth.4285 28481363 PMC5453245

[CR40] Katoh K, Standley DM (2013) MAFFT multiple sequence alignment software version 7: improvements in performance and usability. Mol Biol Evol 30(4):772–780. 10.1093/molbev/mst010 23329690 PMC3603318

[CR41] Kearse M, Moir R, Wilson A, Stones-Havas S, Cheung M, Sturrock S, Buxton S, Cooper A, Markowitz S, Duran C, Thierer T, Ashton B, Meintjes P, Drummond A (2012) Geneious Basic: an integrated and extendable desktop software platform for the organization and analysis of sequence data. Bioinformatics 28(12):1647–1649. 10.1093/bioinformatics/bts199 22543367 PMC3371832

[CR42] Kono A, Umeda-Hara C, Adachi S, Nagata N, Konomi M, Nakagawa T, Uchimiya H, Umeda M (2007) The Arabidopsis D-type cyclin CYCD4 controls cell division in the stomatal lineage of the hypocotyl epidermis. Plant Cell 19(4):1265–1277. 10.1105/tpc.106.046763 17449809 PMC1913761

[CR43] Koornneef M, Meinke D (2010) The development of Arabidopsis as a model plant. Plant J 61(6):909–921. 10.1111/j.1365-313X.2009.04086.x 20409266

[CR44] Kumar N, Larkin JC (2017) Why do plants need so many cyclin-dependent kinase inhibitors? Plant Signal Behav 12(2):e1282021. 10.1080/15592324.2017.1282021 28165885 PMC5351735

[CR45] Lee J-Y, Mummenhoff K, Bowman JL (2002) Allopolyploidization and evolution of species with reduced floral structures in Lepidium L. (Brassicaceae). Proc Natl Acad Sci U S A 99(26):16835–16840. 10.1073/pnas.242415399 12481035 PMC139230

[CR46] Letunic I, Bork P (2024) Interactive Tree of Life (iTOL) v6: recent updates to the phylogenetic tree display and annotation tool. Nucleic Acids Res 52(W1):W78–W82. 10.1093/nar/gkae268 38613393 PMC11223838

[CR47] Li H, Fan Y, Yu J, Chai L, Zhang J, Jiang J, Cui C, Zheng B, Jiang L, Lu K (2018) Genome-wide identification of flowering-time genes in brassica species and reveals a correlation between selective pressure and expression patterns of vernalization-pathway genes in Brassica napus. Int J Mol Sci. 10.3390/ijms19113632 PMC6274771 30453667

[CR48] Lily ZL, Fahlgren N, Kutchan T, Schachtman D, Ge Y, Gesch R, George S, Dyer J, Abdel-Haleem H (2021) Discovering candidate genes related to flowering time in the spring panel of Camelina sativa. Ind Crops Prod 173:114104. 10.1016/j.indcrop.2021.114104

[CR49] Marone D, Russo MA, Laidò G, De Leonardis AM, Mastrangelo AM (2013) Plant nucleotide binding site-leucine-rich repeat (NBS-LRR) genes: active guardians in host defense responses. Int J Mol Sci 14(4):7302–7326. 10.3390/ijms14047302 23549266 PMC3645687

[CR50] Meijer M, Murray JAH (2000) The role and regulation of D-type cyclins in the plant cell cycle. Plant Mol Biol 43(5–6):621–633. 10.1023/a:1006482115915 11089865

[CR51] Meng J, Peng M, Yang J, Zhao Y, Hu J, Zhu Y, He H (2020) Genome-wide analysis of the cyclin gene family and their expression profile in *Medicago truncatula*. Int J Mol Sci 21(24):9430. 10.3390/ijms21249430 33322339 PMC7763586

[CR52] Menges M, Pavesi G, Morandini P, Bögre L, Murray JA (2007) Genomic organization and evolutionary conservation of plant D-type cyclins. Plant Physiol 145(4):1558–1576. 10.1104/pp.107.104901 17951462 PMC2151690

[CR53] Meskiene I, Bögre L, Dahl M, Pirck M, Ha DT, Swoboda I, Heberle-Bors E, Ammerer G, Hirt H (1995) cycMs3, a novel B-type alfalfa cyclin gene, is induced in the G0-to-G1 transition of the cell cycle. Plant Cell 7(6):759–771. 10.1105/tpc.7.6.759 7647566 PMC160829

[CR54] Meyers BC, Kozik A, Griego A, Kuang H, Michelmore RW (2003) Genome-wide analysis of NBS-LRR–encoding genes in Arabidopsis. Plant Cell 15(4):809–834. 10.1105/tpc.009308 12671079 PMC152331

[CR55] Minh BQ, Schmidt HA, Chernomor O, Schrempf D, Woodhams MD, von Haeseler A, Lanfear R (2020) IQ-TREE 2: new models and efficient methods for phylogenetic inference in the genomic era. Mol Biol Evol 37(5):1530–1534. 10.1093/molbev/msaa015 32011700 PMC7182206

[CR56] Mohd Saad NS, Neik TX, Thomas WJW, Amas JC, Cantila AY, Craig RJ, Edwards D, Batley J (2022) Advancing designer crops for climate resilience through an integrated genomics approach. Curr Opin Plant Biol 67:102220. 10.1016/j.pbi.2022.102220 35489163

[CR57] Niu Y, Wu L, Li Y, Huang H, Qian M, Sun W, Zhu H, Xu Y, Fan Y, Mahmood U, Xu B, Zhang K, Qu C, Li J, Lu K (2020) Deciphering the transcriptional regulatory networks that control size, color, and oil content in Brassica rapa seeds. Biotechnol Biofuels 13(1):90. 10.1186/s13068-020-01728-6 32467731 PMC7236191

[CR58] Panchy N, Lehti-Shiu M, Shiu SH (2016) Evolution of gene duplication in plants. Plant Physiol 171(4):2294–2316. 10.1104/pp.16.00523 27288366 PMC4972278

[CR59] Patterson JO, Basu S, Rees P, Nurse P (2021) CDK control pathways integrate cell size and ploidy information to control cell division. eLife 10:e64592. 10.7554/eLife.64592 34114564 PMC8248981

[CR60] Pearson WR (2013) An introduction to sequence similarity (homology) searching. Curr Protoc Bioinf Chap 3:3.1.1–3.1.8. 10.1002/0471250953.bi0301s42 PMC3820096 23749753

[CR61] Perazzolli M, Malacarne G, Baldo A, Righetti L, Bailey A, Fontana P, Velasco R, Malnoy M (2014) Characterization of resistance gene analogues (RGAs) in apple (Malus × domestica Borkh.) and their evolutionary history of the Rosaceae family. PLoS ONE 9(2):e83844–e83844. 10.1371/journal.pone.0083844 24505246 PMC3914791

[CR63] Putterill J, Laurie R, Macknight R (2004) It’s time to flower: the genetic control of flowering time. BioEssays 26(4):363–373. 10.1002/bies.20021 15057934

[CR62] Python Software Foundation (2025) Python Language Reference, version 3.13. https://www.python.org/

[CR64] Qiao X, Li Q, Yin H, Qi K, Li L, Wang R, Zhang S, Paterson AH (2019) Gene duplication and evolution in recurring polyploidization-diploidization cycles in plants. Genome Biol 20(1):38. 10.1186/s13059-019-1650-2 30791939 PMC6383267

[CR66] Rahman M, Hoque A, Roy J (2022) Linkage disequilibrium and population structure in a core collection of *Brassica napus* (L). PLoS ONE 17(3):e0250310. 10.1371/journal.pone.0250310 35231054 PMC8887726

[CR65] Raman H, Raman R, Qiu M, Yadav A, Sureshkumar S, Borg L, Rohan M, Wheeler D, Owen O, Menz I, Balasubramanian S (2019) GWAS hints at pleiotropic roles for FLOWERING LOCUS T in flowering time and yield-related traits in canola. BMC Genomics. 10.1186/s12864-019-5964-y PMC6685183 31387521

[CR67] Razin SV, Ioudinkova ES, Kantidze OL, Iarovaia OV (2021) Co-regulated genes and gene clusters. Genes (Basel) 12(6):907. 10.3390/genes12060907 34208174 PMC8230824

[CR68] Reichheld J-P, Chaubet N, Shen WH, Renaudin JP, Gigot C (1996) Multiple A-type cyclins express sequentially during the cell cycle in *Nicotiana tabacum* BY-2 cells. Proc Natl Acad Sci USA 93:13819–13824. 10.1073/pnas.93.24.13819 8943019 PMC19436

[CR69] Richly E, Kurth J, Leister D (2002) Mode of amplification and reorganization of resistance genes during recent Arabidopsis thaliana evolution. Mol Biol Evol 19(1):76–84. 10.1093/oxfordjournals.molbev.a003984 11752192

[CR70] Romeiro Motta M, Zhao XA, Pastuglia M, Belcram K, Roodbarkelari F, Komaki M, Harashima H, Komaki S, Kumar M, Bulankova P, Heese M, Riha K, Bouchez D, Schnittger A (2022) B1 type cyclins control microtubule organization during cell division in *Arabidopsis*. EMBO Rep 23(1):e53995. 10.15252/embr.202153995 34882930 PMC8728612

[CR71] Roudier F, Fedorova E, Györgyey J, Feher A, Brown S, Kondorosi A, Kondorosi E (2000) Cell cycle function of a Medicago sativa A2-type cyclin interacting with a PSTAIRE‐type cyclin‐dependent kinase and a retinoblastoma protein. Plant J 23(1):73–83 10929103 10.1046/j.1365-313x.2000.00794.x

[CR72] Roudier F, Fedorova E, Lebris M, Lecomte P, Györgyey J, Vaubert D, Horvath G, Abad P, Kondorosi A, Kondorosi E (2003) The Medicago species A2-type cyclin is auxin regulated and involved in meristem formation but dispensable for endoreduplication-associated developmental programs. Plant Physiol 131(3):1091–1103. 10.1104/pp.102.011122 12644661 PMC166874

[CR73] Rousseau-Gueutin M, Belser C, Da Silva C, Richard G, Istace B, Cruaud C, Falentin C, Boideau F, Boutte J, Delourme R, Deniot G, Engelen S, de Carvalho JF, Lemainque A, Maillet L, Morice J, Wincker P, Denoeud F, Chèvre AM, Aury JM (2020) Long-read assembly of the Brassica napus reference genome Darmor-bzh. Gigascience 9(12):giaa137. 10.1093/gigascience/giaa137 33319912 PMC7736779

[CR75] Sankoff D, Zheng C (2018) Whole genome duplication in plants: implications for evolutionary analysis. Methods Mol Biol 1704:291–315. 10.1007/978-1-4939-7463-4_10 29277870

[CR76] Schiessl SV, Huettel B, Kuehn D, Reinhardt R, Snowdon RJ (2017) Flowering time gene variation in brassica species shows evolutionary principles. Front Plant Sci. 10.3389/fpls.2017.01742 PMC5651034 29089948

[CR77] Shi T, Van de Peer Y (2026) Revisiting ancient whole-genome duplications in the seed and flowering plants through the lens of dosage-sensitive genes. Sci Adv 12:eaea9797. 10.1126/sciadv.aea9797 41481725 PMC12758537

[CR78] Song J-M, Guan Z, Hu J, Guo C, Yang Z, Wang S, Liu D, Wang B, Lu S, Zhou R, Xie W-Z, Cheng Y, Zhang Y, Liu K, Yang Q-Y, Chen L-L, Guo L (2020) Eight high-quality genomes reveal pan-genome architecture and ecotype differentiation of Brassica napus. Nat Plants 6(1):34–45. 10.1038/s41477-019-0577-7 31932676 PMC6965005

[CR79] Song X, Wei Y, Xiao D, Gong K, Sun P, Ren Y, Yuan J, Wu T, Yang Q, Li X, Nie F, Li N, Feng S, Pei Q, Yu T, Zhang C, Liu T, Wang X, Yang J (2021) *Brassica carinata* genome characterization clarifies U’s triangle model of evolution and polyploidy in Brassica. Plant Physiol 186(1):388–406. 10.1093/plphys/kiab048 33599732 PMC8154070

[CR80] Takahagi K, Inoue K, Shimizu M, Uehara-Yamaguchi Y, Onda Y, Mochida K (2018) Homoeolog-specific activation of genes for heat acclimation in the allopolyploid grass *Brachypodium hybridum*. Gigascience. 10.1093/gigascience/giy020 PMC5915950 29697823

[CR81] Tamokou JDD, Mbaveng AT, Kuete V (2017) Chap. 8 - antimicrobial activities of African medicinal spices and vegetables. In: Kuete V (ed) Medicinal spices and vegetables from Africa. Academic Press, pp 207–237. 10.1016/B978-0-12-809286-6.00008-X

[CR82] Nagaharu U (1935) Genome analysis in Brassica with special reference to the experimental formation of B. napus and peculiar mode of fertilization. Jpn J Bot 7:389–452

[CR83] Virtanen P, Gommers R, Oliphant TE, Haberland M, Reddy T, Cournapeau D, Burovski E, Peterson P, Weckesser W, Bright J, van der Walt SJ, Brett M, Wilson J, Millman KJ, Mayorov N, Nelson ARJ, Jones E, Kern R, Larson E, Carey CJ, Polat İ, Feng Y, Moore EW, VanderPlas J, Laxalde D, Perktold J, Cimrman R, Henriksen I, Quintero EA, Harris CR, Archibald AM, Ribeiro AH, Pedregosa F, van Mulbregt P (2020) SciPy 1.0 Contributors. SciPy 1.0: fundamental algorithms for scientific computing in Python. Nat Methods 17(3):261–272. 10.1038/s41592-019-0686-2 32015543 PMC7056644

[CR84] Vollrath P, Chawla HS, Alnajar D, Gabur I, Lee H, Weber S, Ehrig L, Koopmann B, Snowdon RJ, Obermeier C (2021) Dissection of quantitative blackleg resistance reveals novel variants of resistance gene Rlm9 in elite Brassica napus. Front Plant Sci 12:749491. 10.3389/fpls.2021.749491 34868134 PMC8636856

[CR85] Wang J, Qiu Y, Cheng F, Chen X, Zhang X, Wang H, Song J, Duan M, Yang H, Li X (2017) Genome-wide identification, characterization, and evolutionary analysis of flowering genes in radish (Raphanus sativus L). BMC Genomics 18(1):981. 10.1186/s12864-017-4377-z 29258434 PMC5738175

[CR86] Warwick SI (2011) Brassicaceae in agriculture. In: Schmidt R, Bancroft I (eds) Genetics and genomics of the Brassicaceae. Plant genetics and genomics: crops and models. Springer, New York, pp 33–65. 10.1007/978-1-4419-7118-0_2

[CR87] Weingartner M, Pelayo HR, Binarova P, Zwerger K, Melikant B, de la Torre C, Heberle-Bors E, Bögre L (2003) A plant cyclin B2 is degraded early in mitosis and its ectopic expression shortens G2-phase and alleviates the DNA-damage checkpoint. J Cell Sci 116(3):487–498. 10.1242/jcs.00250 12508110

[CR88] Wötzel S, Andrello M, Albani MC, Koch MA, Coupland G, Gugerli F (2022) Arabis alpina: a perennial model plant for ecological genomics and life-history evolution. Mol Ecol Resour 22(2):468–486. 10.1111/1755-0998.13490 34415668 PMC9293087

[CR89] Xu L, Hu K, Zhang Z, Guan C, Chen S, Hua W, Li J, Wen J, Yi B, Shen J, Ma C, Tu J, Fu T (2015) Genome-wide association study reveals the genetic architecture of flowering time in rapeseed (Brassica napus L). DNA Res 23(1):43–52. 10.1093/dnares/dsv035 26659471 PMC4755526

[CR90] Yang J, Liu D, Wang X, Ji C, Cheng F, Liu B, Hu Z, Chen S, Pental D, Ju Y, Yao P, Li X, Xie K, Zhang J, Wang J, Liu F, Ma W, Shopan J, Zheng H, Mackenzie SA, Zhang M (2016) The genome sequence of allopolyploid Brassica juncea and analysis of differential homoeolog gene expression influencing selection. Nat Genet 48(10):1225–1232. 10.1038/ng.3657 27595476

[CR91] Yim WC, Swain ML, Ma D, An H, Bird KA, Curdie DD, Wang S, Ham HD, Luzuriaga-Neira A, Kirkwood JS, Hur M, Solomon JKQ, Harper JF, Kosma DK, Alvarez-Ponce D, Cushman JC, Edger PP, Mason AS, Pires JC, Tang H, Zhang X (2022) The final piece of the Triangle of U: evolution of the tetraploid *Brassica carinata* genome. Plant Cell 34(11):4143–4172. 10.1093/plcell/koac249 35961044 PMC9614464

[CR92] Yu Y, Steinmetz A, Meyer D, Brown S, Shen WH (2003) The tobacco A-type cyclin, Nicta;CYCA3;2, at the nexus of cell division and differentiation. Plant Cell 15(11):2763–2777. 10.1105/tpc.015990 14615597 PMC282795

[CR93] Zhang T, Wang X, Lu Y, Cai X, Ye Z, Zhang J (2014) Genome-wide analysis of the cyclin gene family in tomato. Int J Mol Sci 15(1):120–140. 10.3390/ijms15010120 PMC3907801 24366066

[CR94] Zhao B, Zhou M, Ren W, Li H, Zhang Q, He G, Liu Y, He H (2022a) The B-type cyclin CYCB1-1 regulates embryonic development and seed size in maize. Int J Mol Sci 23(11):5907. 10.3390/ijms23115907 35682593 PMC9180882

[CR95] Zhao P, Zhang C, Song Y, Xu X, Wang J, Wang J, Zheng T, Lin Y, Lai Z (2022b) Genome-wide identification, expression and functional analysis of the core cell cycle gene family during the early somatic embryogenesis of *Dimocarpus longan* Lour. Gene 821:146286. 10.1016/j.gene.2022.146286 35176425

[CR96] Zulfiqar S, Zhao T, Liu Y, Wei L, Farooq MA, Tabusam J, Zhao J, Chen X, Wang Y, Xuan S, Li N, Lu Y, Luo S, Shen S, Gu A (2022) Genome-Wide Identification, Characterization, and Transcriptomic Analysis of the Cyclin Gene Family in Brassica rapa. Int J Mol Sci 23(22):14017. 10.3390/ijms232214017 PMC9699369 36430495

